# Supplementary material for: COVID-19 and sexual violence against women: A qualitative study about young people and professionals’ perspectives in Spain
Source: PLoS One. 2023 Aug 2;18(8):e0289402. doi: 10.1371/journal.pone.0289402 (PMC10395938; doi:10.1371/journal.pone.0289402)
Supplement: S1 Table — Consolidated criteria for reporting qualitative research. (DOCX) [file pone.0289402.s001.docx]

S1 Table COREQ CHECKLIST. Consolidated criteria for reporting qualitative research

| No. Item | Guide questions/description | Reported |
| --- | --- | --- |
| Domain 1: Research team an reﬂexivity | | |
| 1. Inter viewer/facilitator | Which author/s conducted the  interview? | The interviews were conducted by three interviewers from the research team with training and experience in qualitative research methodology |
| 2. Credentials | What were the researcher’s credentials? |  |
| 3. Occupation | What was their occupation at the time of the study? |  |
| 4. Gender | Was the researcher male or female? | The interviews were conducted by one male and two female researchers.  Gender criteria were considered for conducting interviews with young people: women interviewed girls and the male interviewer interviewed boys due to the youth of the population to be interviewed and the sensitivity of the topic addressed, trying to guarantee rapport and the establishment of trust to achieve the fluidity of the discourse. The development of interviews with professionals was not guided by these criteria. |
| 5. Experience and training | What experience or training did the researcher have? | Interviewers have sufficient training and experience in qualitative research methodology |
| 6. Relationship with participants established | Was a relationship established prior to  study commencement? | No |
| 7. Participant knowledge  of the interviewer | What did the participants know about  the researcher? | The study participants were informed at the time of contact and at the beginning of the interviews, about the project, the research leader and the university responsible of it, their scope and study objectives. |
| 8. Interviewer  characteristics | What characteristics were reported  about the inter viewer/facilitator? |  |
| Domain 2: study design | | |
| 9. Methodological  orientation and Theory | What methodological orientation was  stated to underpin the study? | Content analysis |
| 10. Sampling | How were participants selected? | Participants were identified, selected and recruited by an initial online prospection of potential participants, dissemination of the study online and following a snowball strategy |
| 11. Method of approach | How were participants approached? | An initial email was sent to each of them to invite them to participle. In some cases, we reinforce this initial contact by telephone. |
| 12. Sample size | How many participants were in the study? | 23 young people were interviewed: 11 women, 12 men  16 professionals were interviewed: 13 women and 3 men, from youth services and sexual violence against women. |
| 13. Non-­‐participation | How many people refused to participate or dropped out? Reasons? | This information was not recorded. |
| 14. Setting of data collection | Where was the data collected? | Interviews were carried out by telephone and video call |
| 15. Presence of non-­‐  participants | Was anyone else present besides the  participants and researchers? | No |
| 16. Description of sample | What are the important characteristics of the sample? | For the selection of professionals, the following factors were considered: 1) type of service: public administration and third sector entities; 2) scope of action: SV attention and prevention activities and/or youth services; and 3) geographical distribution by Spanish regions. For the selection of young people, the following variables were also considered: 1) age (18-21 years and 22 to 24 years to achieve a balanced representation); (2) women and men; 3) level of studies (university and non-university); (4) country of origin (Spain and others); 5) geographical distribution by Spanish regions. |
| 17. Interview guide | Were questions, prompts, guides provided by the authors? | Interview guide was piloted and adapted in the first phases of the field work in joint work sessions with different members of the research team |
| 18. Repeat interviews | Were repeat interviews carried out? | No |
| 19. Audio/visual recording | Did the research use audio or visual recording to collect the data? | Audio recording was used to collect data from telephone and video call interviews |
| 20. Field notes | Were ﬁeld notes made during and/or after the interview? | Field notes were recorded at the end of each interview, making difference: a) information from the context of the interview and communication, b) principle ideas emerged from the discourse. Those were used by the analysts to approach the interviews. |
| 21. Duration | What was the duration of the interviews? | The duration of the interviews was from 50 to 70 minutes |
| 22. Data saturation | Was data saturation discussed? | The research team considered that data saturation was achieved when latest interviews did not generate new additional information. |
| 23. Transcripts returned | Were transcripts returned to  participants for comment and/or correction? | No |
| Domain 3: analysis and ﬁndings | | |
| 24. Number of data coders | How many data coders coded the data? | One analyst from the research team |
| 25. Description of the  coding tree | Did authors provide a description of the  coding tree? | No, because the coding tree was not used for analysis. |
| 26. Derivation of themes | Were themes identiﬁed in advance or derived from the data? | No previous themes were identified in the analysis.  First, a pre-analysis was carried out to systematise and organise the information accord-ing to the dimensions of the interview script. Those questions related to the impact of the pandemic were narrowed down to create an initial structure of categories and codes. The codes were grouped according to their discursive similarity and divergence with each informant profile; the emerging topics that were constructed explained the elements that impacted SV during confinement, considering the context from which each informant spoke (professionals and young people). |
| 27. Software | What software, if applicable, was used to manage the data? | No software was used |
| 28. Participant checking | Did participants provide feedback on the ﬁndings? | Because of the overload work of the professionals from IPV-response services we decide not to recontact them, so feedback was not collected. |
| 29. Quotations presented | Were participant quotations presented to illustrate the themes/ﬁndings? Was  each quotation identiﬁed? | Identified quotations are added in the manuscript in results section |
| 30. Data and ﬁndings consistent | Was there consistency between the data presented and the ﬁndings? | Yes |
| 31. Clarity of major themes | Were major themes clearly presented in the ﬁndings? | Yes |
| 32. Clarity of minor  themes | Is there a description of diverse cases or discussion of minor themes? | Yes |
