# Supplementary material for: COVID-19 and sexual violence against women: A qualitative study about young people and professionals’ perspectives in Spain
Source: PLoS One. 2023 Aug 2;18(8):e0289402. doi: 10.1371/journal.pone.0289402 (PMC10395938; doi:10.1371/journal.pone.0289402)
Supplement: S2 Table — COVID-19 and sexual violence against women: A qualitative study about young people and professionals’ perspectives in Spain. (DOCX) [file pone.0289402.s002.docx]

Annex.2 Coding tree

COVID-19 and sexual violence against women: A qualitative study about young people and professionals’ perspectives in Spain

| Themes | Categories | Codes |
| --- | --- | --- |
| Impact on professionals’ answers | **Services-related difficulties** | The prompt paralysis and suspension |
|  |  | Alternatives are sought |
|  |  | Adapting to the context of the pandemic |
|  |  | Handicaps of on-line care |
|  |  | Limitations on-line care and follow-up |
|  |  | Impact on primary prevention |
|  |  | Limited detection readiness |
|  | **Oportunities** | How affected women may find advantages in online attention |
|  |  | Advantages for minority young women. |
|  |  | Advantages related services |
|  |  | Advantages related professionals |
|  | **Victims-related challenges** | SV cases among minority social groups |
| Impact on sexual violence | **Increased SV** | Intimate Sexual Violence |
|  |  | Family setting |
|  |  | SV in digital setting |
|  | **Decreased SV** | Non-partner sexual violence |
|  |  | Public settings |
|  |  | Discrepancies by youth sex |
|  |  | Positive impact of COVID-19 lockdown |
|  |  | Professionals’ experience |
|  | **Silenced/ unknowledge** | Intimate partner sexual violence |
|  |  | Close cases |
|  |  | Family setting |
|  |  | Limited access formal services |
|  |  | Victims’ isolation and fear |
