# Supplementary material for: COVID-19 and sexual violence against women: A qualitative study about young people and professionals’ perspectives in Spain
Source: PLoS One. 2023 Aug 2;18(8):e0289402. doi: 10.1371/journal.pone.0289402 (PMC10395938; doi:10.1371/journal.pone.0289402)
Supplement: Attachment — Submitted filename: Response to reviewers.docx [file pone.0289402.s003.docx]

PONE-D-22-16766

COVID-19 and sexual violence against women: A qualitative study about young people and professionals’ perspectives in Spain.

PLOS ONE
Dear Dr. Carmen Vives
Thank you for submitting your manuscript to PLOS ONE. After careful consideration, we feel that it has merit but does not fully meet PLOS ONE’s publication criteria as it currently stands. Therefore, we invite you to submit a revised version of the manuscript that addresses the points raised during the review process.
Even though we have to very different positions regarding the current paper, I agree with reviewer 2 in the sense that there is much work to be done. In its current condition this paper cannot be published. I will not reject the paper but provide the authors the opportunity to respond to comments and work on the document. I have other suggestions and comments.
As reviewer 2 says, the qualitative analysis is extremely descriptive and does not provide a theory under the social sciences nor methodological orientation that underpinned the study. Was it guided by grounded theory, discourse analysis, ethnography, phenomenology, or content analysis? This point is fundamental to analyzing and presenting the results of their work.

**The data analyses section has been re-written in order to improve the accuracy of the information provided. Please see page 8 that includes the following information:**

A qualitative content analysis was carried out using predefined tree codes that were agreed upon by the research team, based on the study objectives and a preliminary analysis of information from the interviews. After using this initial tree of codes, new codes that emerged were added during the process of analysis process. The tree of codes was adapted during the coding process, by unifying codes according to their discursive similarity and divergence with each informant profile; the emerging topics that were constructed explained the elements that impacted SV during confinement, considering the context from which each informant spoke (professionals and young people). In order to prevent possible bias in coding, we carried out these the following measures were used: double coding of interviews, contrasts of the usefulness of the emerging codes among the analysts and debate sessions with the rest of the members of the research team. The analysis was carried out manually.

I suggest that the authors to incorporate elements from the model into the main text; for instance, what experience and training do the researchers enjoy? What information did participants have about the researchers, e.g., their personal goals and reasons for carrying out the study? How many people refused to participate, how many dropped out, and why? What are the main limitations and strengths of the study? Having say this, I suggest the authors to work hard on this study and resolve the different issues in order to have a better version of the document.

**As observed on pages 5-7 of the current version of the manuscript, we provided more information about participants’ recruitment procedure and ethical-related considerations in the following terms:**

Participants (page 5)

The selection criteria for the young people were: 1) age (18-21 years and 22-24 years); 2) sex (women/men); 3) level of studies (university or not); 4) place of origin (migrant and national); and 5) geographical distribution according to region. To select the professionals, the following characteristics of the resources where they worked were considered: 1) type of resource management (public administration or associations); 2) scope of action (approach to SV and youth services); and 3) geographical distribution according to the considered regions (Valencia, the Balearic Islands, Catalonia, Madrid, the Basque Country and Navarre). All these last potential contacts were identified through their institution’s webpages and/or previous contacts of the research team. In the case of young people, through advertisements and flyers distributed in educational environments (high schools, universities, etc.), social media online platforms (Facebook, Instagram, etc.), non-governmental organizations (NGOs) and state institutions working with young people. The criteria to finalize information collection was discourse saturation. Finally, 23 young people and 15 professionals were interviewed.

Ethical considerations (page 6 and 7).

A protocol was implemented to guarantee the ethical principles of the research and the General Data Protection Regulation (GDPR). The study was conducted in accordance with the Declaration of Helsinki and approved by the Ethical Committee of the University of Alicante (UA-2020-07-07). Due to COVID-19 mobility restrictions, interviews were conducted by phone or video call, and therefore, written consent could not be obtained. The Institutional Review Board approved use of oral consent. Documentation on the study was provided and informed consent was given prior to the interviews. In this sense, a paragraph was read at the beginning of the interviews to inform the subjects about the project and the conditions of participating in it, as well as requesting verbal consent from them.

**We also rewrote results section in order to provide more accurate information according to participants’ profile and its context.**

**Authors’ response to reviewers**

Reviewer #1: It's a valuable manuscript and reads fluently. I congratulate the authors on the use of this qualitative approach.
The structure of the article is very good and clear.
I have not doubts about the methodology and the discussion.

**Thank you for your comment.**

Reviewer #2: Even though the research theme is quite important and interesting, there are several issues that will imply a design change of the study and a major revision. I list the following:

1. Number of authors is excessive, there is no way that 10 persons have contribute to write, analyse and discuss information. probably some just did filed work and interviewed participants or transcribed the data.

**This manuscript is part of a large project aimed at analyzing the magnitude, associated factors and perceptions of sexual violence in a sample of young people and stakeholders in different regions of Spain. The authors of this paper make up the research team involved in the project. In the current version of the manuscript, we have revised and improved the information about the authors’ contributions to the papers in the following terms:**

| **Conceptualization** | Carmen Vives-Cases, Esther Castellanos-Torres, Belén Sanz-Barbero contributed altogether to the first ideas of this study and Laura Otero-García, Maria José López, Gloria Pérez, Gemma Renart-Vicens, Carmen Saurina, Laura Serra, and Laura Vall-Llosera Casanovas made relevant contributions to achieve the final formulation of the aim of this study. |
| --- | --- |
| **Data Curation** | Esther Castellanos-Torres and Carmen Vives-Cases proposed the initial codes and later categories based on the different readings of the transcriptions. Laura Otero-García and Belen Sanz-Barbero helped us in case of discrepancies. |
| **Formal Analysis** | Esther Castellanos-Torres and Laura Otero-García worked on this part with the supervision of Carmen Vives-Cases and Belen Sanz-Barbero. |
| **Funding Acquisition** | Belen Sanz-Barbero, Carmen Vives-Cases, Laura Otero-García, Maria José López, Gloria Pérez, Gemma Renart-Vicens, Carmen Saurina, Laura Serra, Laura Vall-Llosera Casanovas got financial support for the project leading to this publication. |
| **Investigation** | Conducting a research and research process, specifically performing the experiments, or data/evidence collection. |
| **Methodology** | Belen Sanz-Barbero, Carmen Vives-Cases, Laura Otero-García, Maria José López, Gloria Pérez, Gemma Renart-Vicens, Carmen Saurina, Laura Serra, and Laura Vall-Llosera Casanovas designed the scripts, participant selection criteria and data recruitment strategies. Esther Castellanos-Torres coordinated data collection through personal interviews. |
| **Project Administration** | Carmen Vives-Cases and Belen Sanz-Barbero as PI of the project. |
| **Resources** | Laura Otero-García, Maria José López, Gloria Pérez, Gemma Renart-Vicens, Carmen Saurina, Laura Serra, and Laura Vall-Llosera Casanovas provided contacts with different institutions related to sexual violence and violence against women prevention to identify potential participants. |
| **Software** | Not applicable. |
| **Supervision** | Carmen Vives-Cases and Belen Sanz-Barbero as PI of the project. |
| **Validation** | Not applicable. |
| **Visualization** | Carmen Vives-Cases and Belen Sanz-Barbero as PI of the project. |
| **Writing – Original Draft Preparation** | Esther Castellanos-Torres, Carmen Vives-Cases and Belen Sanz-Barbero as PI of the project. |
| **Writing – Review & Editing** | Laura Otero-García, Maria José López, Gloria Pérez, Gemma Renart-Vicens, Carmen Saurina, Laura Serra, and Laura Vall-Llosera Casanovas contributed to the drafts until they approved the final version. |

2. Selection of participants was done through snow ball procedure, which may have skewed the opinions and it is the least rigorous qualitative research design.

**As observed on page 5 of the current version of the manuscript, we provided more information about participants’ recruitment procedure in the following terms:**

The selection criteria for the young people were: 1) age (18-21 years and 22-24 years); 2) sex (women/men); 3) level of studies (university or not); 4) place of origin (migrant and national); and 5) geographical distribution according to region. To select the professionals, the following characteristics of the resources where they worked were considered: 1) type of resource management (public administration or associations); 2) scope of action (approach to SV and youth services); and 3) geographical distribution according to the considered regions (Valencia, the Balearic Islands, Catalonia, Madrid, the Basque Country and Navarre). All these last potential contacts were identified through their institution’s webpages and/or previous contacts of the research team. In the case of young people, through advertisements and flyers distributed in educational environments (high schools, universities, etc.), social media online platforms (Facebook, Instagram, etc.), non-governmental organizations (NGOs) and state institutions working with young people. The criteria to finalize information collection was discourse saturation. Finally, 23 young people and 15 professionals were interviewed.

3. Referring to results, there are not substantial contributions and several of the findings are obvious, such as that violence in public spaces would be reduced due to confinement and domestic violence or in private spaces would increase, or that digital violence against women increased,

**The main contribution of this study is putting together professionals’ and young people’s perceptions about the impact of the COVID-19 lockdown in a prevalent issue which is sexual violence against women and men of different sexual orientation. As explained in the introduction of the manuscript (see page 4):**

Despite the valuable contribution made by the testimonies from professionals on the front line in terms of this type of violence, there are still few studies that contrast such professional perceptions with those of women [23]. Qualitative studies have also revealed how COVID-19 preventive measures and the economic crisis hindered and strained intimate relationships among young people, although previous studies focusing on their perspective are also limited [24,25].

**The information provided in the paper may contribute to future strategies to cope with this issue in other critical periods as explained with specific implications in the conclusion (page 27):**

From a perspective of participating professionals in this study, the confinement and prevention measures for COVID-19 have a negative impact on attending to and monitoring SV cases. The need to develop protocols for action around violence against women and SV in crisis contexts emerges, as well as the need to improve accessibility to these resources, guaranteeing, for example, their continuity during future. Young people and professionals agree in the fact that there has been a decrease in cases in public spaces due to confinement and social isolation, although there has been a possible continuity through other means, such as social networks, finding SV in online pornography. Their opinions are different regarding the impact of confinement within relationships, mainly due to the lack of knowledge of this problem among those who are younger. It is verified that in the imaginary of young men, SV is associated with the public space and the lack of awareness that SV also occurs in the private space and within relationships, where certain sexist SV practices are naturalized. In this sense, investment in public feminist policies and awareness strategies are required in terms of all kinds of SV against women.

**In addition, in the current version of the manuscript, we have rewritten the results section in order to provide more accurate information according to participant profiles and contexts.**

4. Several of the testimonials are very short and the context is not fully understood.

**Thank you very much for this suggestion. The current version of the manuscript provides many of the selected verbatims to improve the information about the context.**

5. One would think that with 39 interviews and having interviewed young people and professionals there would be much more information and summarizing in 4 categories probably leaves out other relevant information, especially when we read the issues that were explored in the interviews. This is probably because only one person coded the interview information when it is always better to have more people involved in the coding.

**Data analyses section had been re-written in order to improve the accuracy of the information provided. Please see page 8 the following information:**

A qualitative content analysis was carried out using predefined tree codes that were agreed upon by the research team, based on the study objectives and a preliminary analysis of information from the interviews. After using this initial tree of codes, new codes that emerged were added during the process of analysis process. The tree of codes was adapted during the coding process, by unifying codes according to their discursive similarity and divergence with each informant profile; the emerging topics that were constructed explained the elements that impacted SV during confinement, considering the context from which each informant spoke (professionals and young people). In order to prevent possible bias in coding, we carried out these the following measures were used: double coding of interviews, contrasts of the usefulness of the emerging codes among the analysts and debate sessions with the rest of the members of the research team. The analysis was carried out manually.

6. Although interviewing both social actors could be a success, there is no integration of what was found because in one case it is about real experiences (those of professionals) and in the other about what the young people imagine.

**Thank you for your comment. As we explained in limitations (page 26):** Most young people who were interviewed have provided their perceptions on the impact on SV without having any experience or knowing anyone in this kind of situation. However, their perceptions seem to be in line with what has been observed in other studies.

**We understand the reviewer’s point, however, we think that it is important to show the different points of view, as they that allow us to compare the perceptions of young people with the experiences of the professionals**

7. Perhaps it would be better to write an article about what professionals observed and changed in the prevention and care of violence against women and focus not only in the barriers but highlight what was learned and what was gained during the pandemic, and another article focused on what young people perceived about the situation, although this seems the weakest information, at least as presented here.

**As we explain in the discussion section,** the information has been triangulated by selecting participants with different profiles (professionals and young people). In addition, the results are supported by different verbatim citations provided by the heterogeneity of participants in our study. The added value of doing that is by identifying common perceptions despite this heterogeneity, as well as differences between professionals and young people and girls and boys.
